# Supplementary material for: Opposing gene regulatory programs governing myofiber development and maturation revealed at single nucleus resolution
Source: Nat Commun. 2023 Jul 19;14:4333. doi: 10.1038/s41467-023-40073-8 (PMC10356771; doi:10.1038/s41467-023-40073-8)

Supplementary Information

Opposing gene regulatory programs governing myofiber development and maturation revealed at single nucleus resolution

Matthieu Dos Santos et al.,

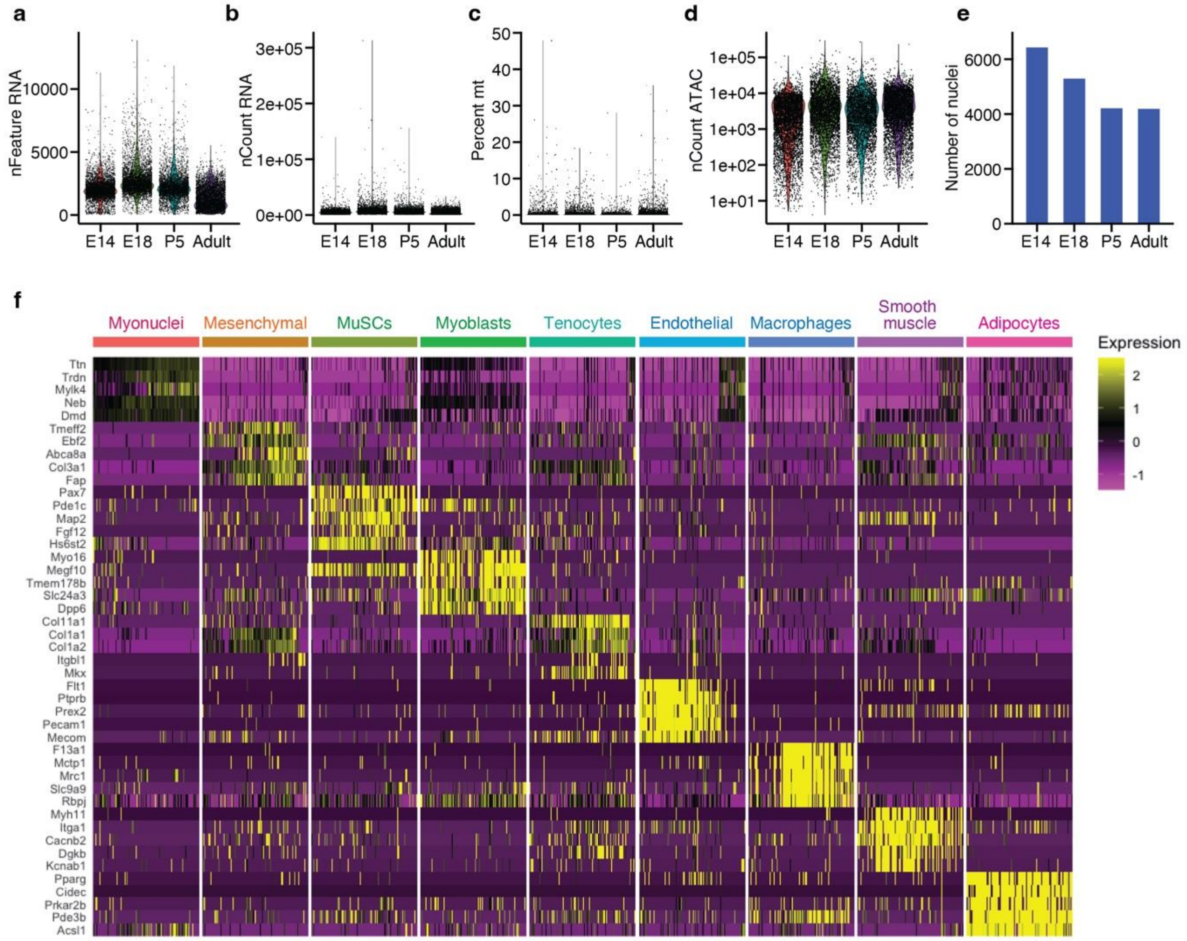

Supplementary Fig. 1: Quality controls and identity of the nuclei populations from snRNA-seq and snATAC-seq experiments from Fig. 1.

(a) Number of detected genes per nucleus in snRNA-seq for each sample. (b) Number of reads per nucleus in snRNA-seq for each sample. (c) Percentage of mitochondrial genes per nucleus in snRNA-seq for each sample. (d) Number of reads per nucleus in snATAC-seq for each sample. (e) Number of nuclei per sample after filtering nuclei that had nCount RNA between 25000 and 400, less than 10% mitochondrial counts, and nCount ATAC between 70000 and 400. (f) Heatmap of the five most expressed genes in each population of cells from snRNA-seq in Fig. 1b (upregulated in yellow and downregulated in violet). The values correspond to z-scores of normalized counts.

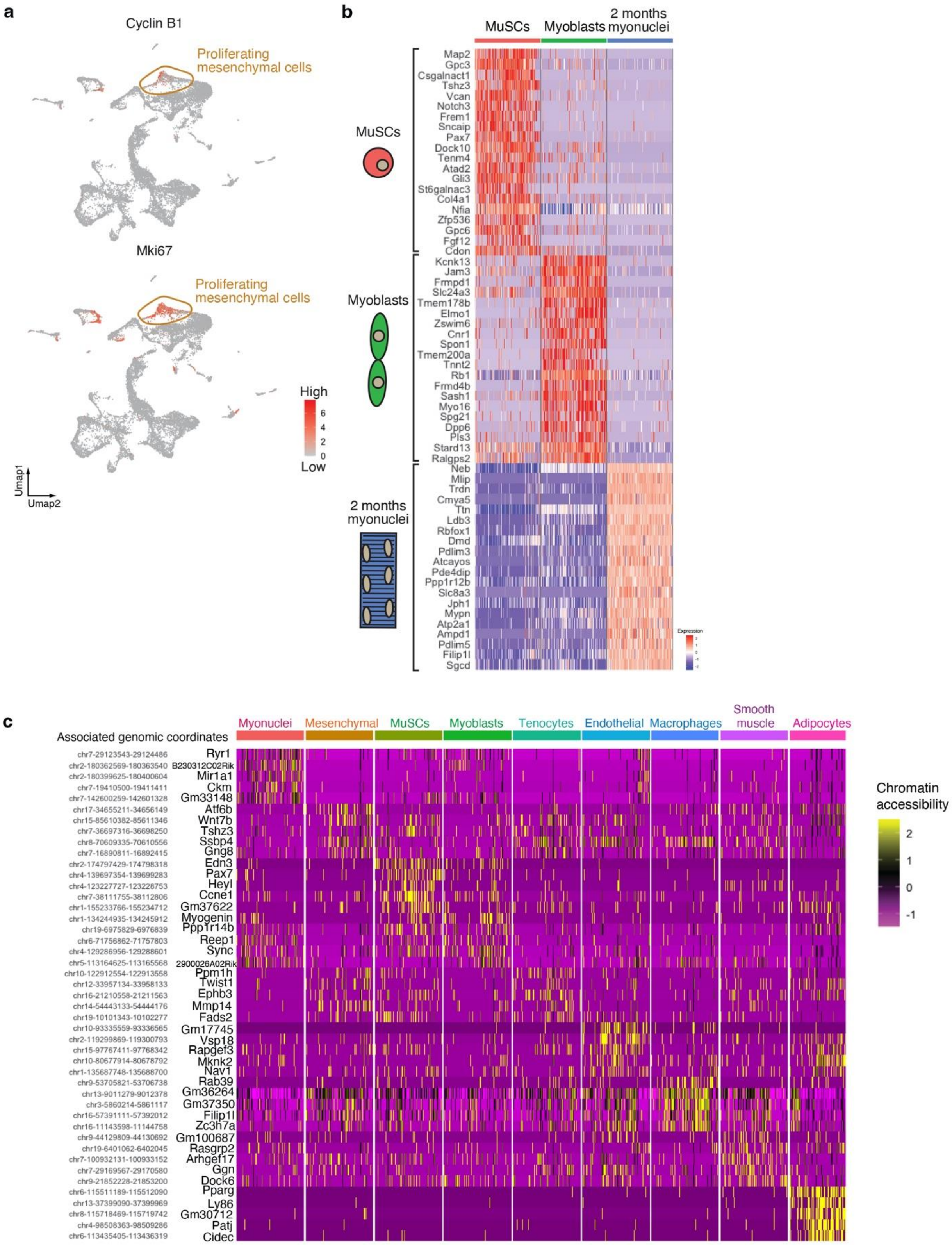

a
Cyclin B1
Proliferating mesenchymal cells
Mki67
Proliferating mesenchymal cells
High
6
4
2
0
Low
Umap1
Umap2
b
MuSCs
Myoblasts
2 months myonuclei
MuSCs
Myoblasts
2 months myonuclei
Map2
Gpc3
Csgalnact1
Tshz3
Vcan
Notch3
Frem1
Sncaip
Pax7
Dock10
Tenm4
Atad2
Gli3
St6galnac3
Col4a1
Nfia
Zfp536
Gpc6
Fgf12
Cdon
Kcnk13
Jam3
Frmpd1
Slc24a3
Tmem178b
Elmo1
Zswim6
Cnr1
Spon1
Tmem200a
Tnnt2
Rb1
Frmd4b
Sash1
Myo16
Spg21
Dpp6
Pls3
Stard13
Ralgps2
Neb
Mlip
Trdn
Cmya5
Ttn
Ldb3
Rbfox1
Dmd
Pdlim3
Atcayos
Pde4dip
Ppp1r12b
Slc8a3
Jph1
Mypn
Atp2a1
Ampd1
Pdlim5
Filip1l
Sgcd
Expression
c
Associated genomic coordinates
Myonuclei
Mesenchymal
MuSCs
Myoblasts
Tenocytes
Endothelial
Macrophages
Smooth muscle
Adipocytes
chr7-29123543-29124486 Ryr1
chr2-180362569-180363540 B230312C02Rik
chr2-180399625-180400604 Mir1a1
chr7-19410500-19411411 Ckm
chr7-142600259-142601328 Gm33148
chr17-34655211-34656149 Atf6b
chr15-85610382-85611346 Wnt7b
chr7-36697316-36698250 Tshz3
chr8-70609335-70610556 Ssbp4
chr7-16890811-16892415 Gng8
chr2-174797429-174798318 Edn3
chr4-139697354-139699283 Pax7
chr4-123227727-123228753 Heyl
chr7-38111755-38112806 Ccne1
chr1-155233766-155234712 Gm37622
chr1-134244935-134245912 Myogenin
chr19-6975829-6976839 Ppp1r14b
chr6-71756862-71757803 Reep1
chr4-129286956-129288601 Sync
chr5-113164625-113165568 2900026A02Rik
chr10-122912554-122913558 Ppm1h
chr12-33957134-33958133 Twist1
chr16-21210558-21211563 Ephb3
chr14-54443133-54444176 Mmp14
chr19-10101343-10102277 Fads2
chr10-93335559-93336565 Gm17745
chr2-119299869-119300793 Vsp18
chr15-97767411-97768342 Rapgef3
chr10-80677914-80678792 Mknk2
chr1-135687748-135688700 Nav1
chr9-53705821-53706738 Rab39
chr13-9011279-9012378 Gm36264
chr3-5860214-5861117 Gm37350
chr16-57391111-57392012 Filip1l
chr16-11143598-11144758 Zc3h7a
chr9-44129809-44130692 Gm100687
chr19-6401062-6402045 Rasgrp2
chr7-100932131-100933152 Arhgef17
chr7-29169567-29170580 Ggn
chr9-21852228-21853200 Dock6
chr6-115511189-115512090 Pparg
chr13-37399090-37399969 Ly86
chr8-115718469-115719742 Gm30712
chr4-98508363-98509286 Patj
chr6-113435405-113436319 Cidec
Chromatin accessibility
2
1
0
-1

Supplementary Fig. 2: Identification of genes and transcription factors regulating myogenesis, and identity of the nuclei populations from snATAC-seq.

(a) The same UMAP visualization as in Fig. 1b, showing the expression of proliferative markers *Cyclin B1* and *Mki67* in proliferating mesenchymal cells. (b) Heatmap of the most differentially expressed genes in MuSCs compared to myoblasts and adult myonuclei (upregulated in red and downregulated in blue). The values correspond to z-scores of normalized counts. (c) Heatmap showing activities of the five top enriched open chromatin peaks and their associated genomic coordinates for each cell cluster from snATAC-seq.

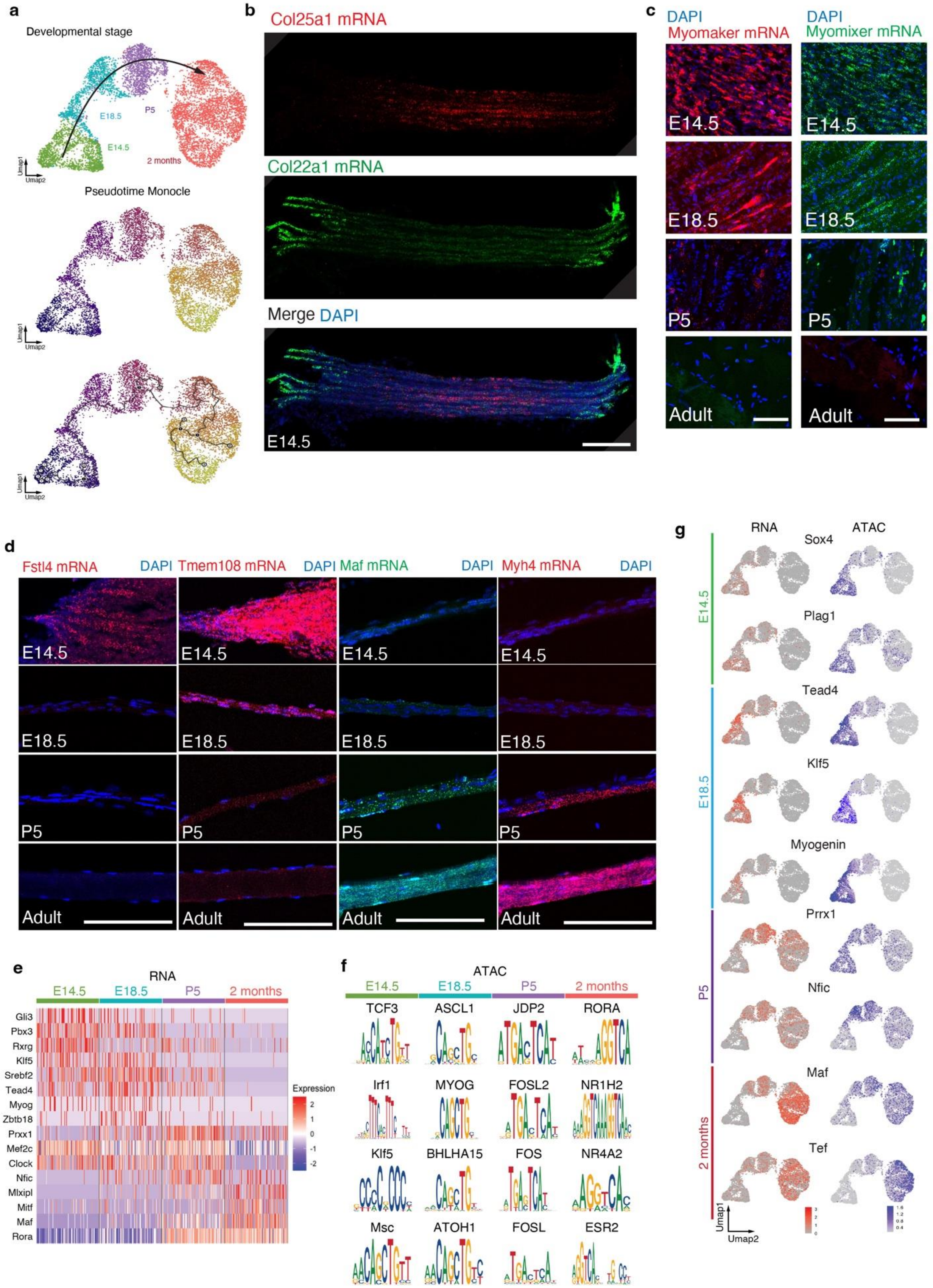
a
Developmental stage
E18.5
P5
E14.5
2 months
Umap1
Umap2
Pseudotime Monocle
b
Col25a1 mRNA
Col22a1 mRNA
Merge DAPI
E14.5
c
DAPI
Myomaker mRNA
DAPI
Myomixer mRNA
E14.5
E18.5
P5
Adult
d
Fstl4 mRNA DAPI
Tmem108 mRNA DAPI
Maf mRNA DAPI
Myh4 mRNA DAPI
E14.5
E18.5
P5
Adult
e
RNA
E14.5
E18.5
P5
2 months
Gli3
Pbx3
Rxrg
Klf5
Srebf2
Tead4
Myog
Zbtb18
Prrx1
Mef2c
Clock
Nfic
Mlxipl
Mitf
Maf
Rora
Expression
f
ATAC
E14.5
E18.5
P5
2 months
TCF3
ASCL1
JDP2
RORA
Irf1
MYOG
FOSL2
NR1H2
Klf5
BHLHA15
FOS
NR4A2
Msc
ATOH1
FOSL
ESR2
g
RNA
ATAC
Sox4
Plag1
Tead4
Klf5
Myogenin
Prrx1
Nfic
Maf
Tef
E14.5
E18.5
P5
2 months
Umap1
Umap2

Supplementary Fig. 3: Identification of transcription factors regulating myonuclei maturation.

(a) The same UMAP visualization as in Fig. 2b, showing the pseudotime trajectory analysis of our data with Monocle software. (b) Single-molecule RNA-FISH (smRNA-FISH) experiments on isolated fibers from E14.5 hindlimb skeletal muscle to visualize *Col22a1* (green), and *Col25a1* (red) mRNAs (representative results from 3 independent experiments). Scale bar: 100µm. (c) SmRNA-FISH experiments on longitudinal muscle fibers sections from E14.5, E18.5, P5, and adult hindlimb skeletal muscle to visualize *Mymomaker* (red), and *Mymomixer* (green) mRNAs (representative results from 3 independent experiments). Scale bar: 100µm. (d) SmRNA-FISH experiments on isolated fibers from E14.5, E18.5, P5, and adult hindlimb skeletal muscle to visualize *Fstl4* (red), *Tmem108* (red), *Maf* (green), and *Myh4* (red) mRNAs (representative results from 3 independent experiments). Scale bar: 100µm. (e) Heatmap of the four most enriched TFs in embryonic, fetal, neonatal, and adult myonuclei from snRNA-seq (upregulated in red and downregulated in blue). The values correspond to z-scores of normalized counts. (f) Position weight matrix (PWM) motifs showing the enrichment of TF motifs in differential peaks in embryonic, fetal, neonatal, and adult myonuclei. (g) UMAPs depicting the expression and activity of the binding motif of Sox4 and Plag1 in embryonic myonuclei (E14.5), Tead4, Klf5 and Myogenin in fetal myonuclei (E18.5), Prrx1 and Nfic in neonatal myonuclei (P5), Maf and Tef in adult myonuclei (2 months).

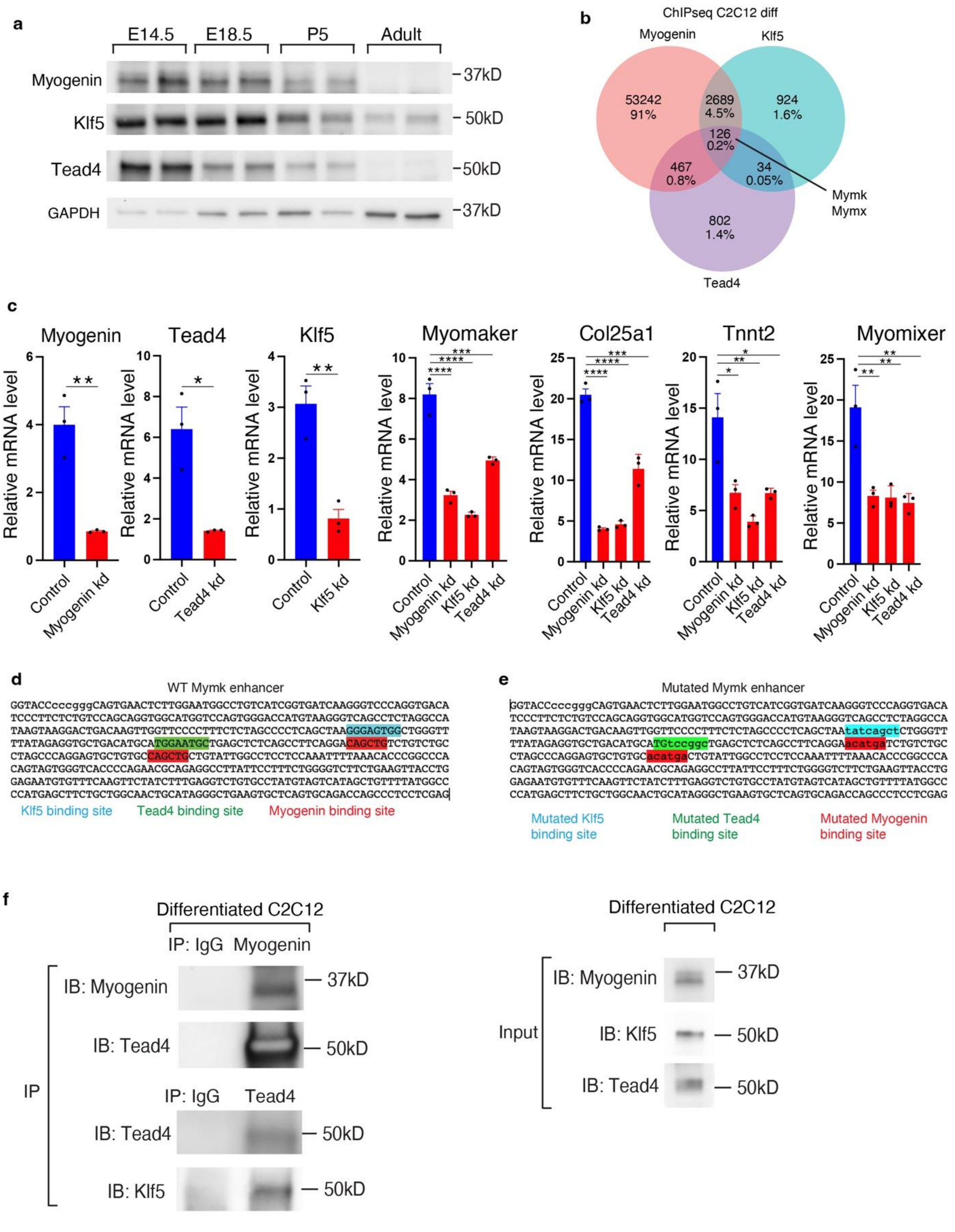

a
E14.5
E18.5
P5
Adult
Myogenin
Klf5
Tead4
GAPDH
37kD
50kD
50kD
37kD
b
ChIPseq C2C12 diff
Myogenin
Klf5
Tead4
53242
91%
2689
4.5%
924
1.6%
126
0.2%
467
0.8%
34
0.05%
802
1.4%
Mymk
Mymx
c
Myogenin
Tead4
Klf5
Myomaker
Col25a1
Tnnt2
Myomixer
Relative mRNA level
Control
Myogenin kd
Tead4 kd
Klf5 kd
d
WT Mymk enhancer
GGTACCcccgggCAGTGAACTCTTGGAATGGCCTGTCATCGGTGATCAAGGGTCCCAGGTGACA
TCCCTTCTCTGTCCAGCAGGTGGCATGGTCCAGTGGGACCATGTAAGGGTCAGCCTCTAGGCCA
TAAGTAAGGACTGACAAGTTGGTTCCCCTTTCTCTAGCCCCTCAGCTAAGGGAGTGGCTGGGTT
TTATAGAGGTGCTGACATGCATGGAATGCTGAGCTCTCAGCCTTCAGGACAGCTGTCTGTCTGC
CTAGCCCAGGAGTGCTGTGCCAGCTGCTGTATTGGCCTCCTCCAAATTTTAAACACCCGGCCCA
CAGTAGTGGGTCACCCCAGAACGCAGAGGCCTTATTCCTTTCTGGGGTCTTCTGAAGTTACCTG
GAGAATGTGTTTCAAGTTCTATCTTTGAGGTCTGTGCCTATGTAGTCATAGCTGTTTTATGGCC
CCATGAGCTTCTGCTGGCAACTGCATAGGGCTGAAGTGCTCAGTGCAGACCAGCCCTCCTCGAG
Klf5 binding site
Tead4 binding site
Myogenin binding site
e
Mutated Mymk enhancer
GGTACCcccgggCAGTGAACTCTTGGAATGGCCTGTCATCGGTGATCAAGGGTCCCAGGTGACA
TCCCTTCTCTGTCCAGCAGGTGGCATGGTCCAGTGGGACCATGTAAGGGTCAGCCTCTAGGCCA
TAAGTAAGGACTGACAAGTTGGTTCCCCTTTCTCTAGCCCCTCAGCTAAtatcagctCTGGGTT
TTATAGAGGTGCTGACATGCATGtccggcTGAGCTCTCAGCCTTCAGGAacatgaTCTGTCTGC
CTAGCCCAGGAGTGCTGTGCacatgaCTGTATTGGCCTCCTCCAAATTTTAAACACCCGGCCCA
CAGTAGTGGGTCACCCCAGAACGCAGAGGCCTTATTCCTTTCTGGGGTCTTCTGAAGTTACCTG
GAGAATGTGTTTCAAGTTCTATCTTTGAGGTCTGTGCCTATGTAGTCATAGCTGTTTTATGGCC
CCATGAGCTTCTGCTGGCAACTGCATAGGGCTGAAGTGCTCAGTGCAGACCAGCCCTCCTCGAG
Mutated Klf5 binding site
Mutated Tead4 binding site
Mutated Myogenin binding site
f
Differentiated C2C12
IP: IgG Myogenin
IB: Myogenin
IB: Tead4
IP
IP: IgG Tead4
IB: Tead4
IB: Klf5
37kD
50kD
50kD
50kD
Differentiated C2C12
Input
IB: Myogenin
IB: Klf5
IB: Tead4
37kD
50kD
50kD

Supplementary Fig. 4: Myogenin, Klf5, and Tead4 activate the expression of developmental muscle genes synergistically.

(a) Western blot showing the expression of Myogenin, Klf5, and Tead4 proteins in embryonic (E14.5), fetal (E18.5), post-natal (P5), and adult WT hindlimb skeletal muscle (n=2). GAPDH is a loading control. (b) Venn diagram of the co-occupied peaks by Myogenin, Tead4, and Klf5 in ChIP-seq data from differentiated C2C12 myotubes. (c) Expression level by RT-qPCR of *Myogenin*, *Klf5*, *Tead4*, *Myomaker*, *Col25a1*, *Tnnt2*, and *Myomixer* in C2C12 infected with shRNA against a control scramble, or *Myogenin*, *Tead4*, and *Klf5* (n=3 independent experiments for each group). (d) DNA sequence of *Mymk* enhancer. Binding sites for Myogenin, Tead4, and Klf5 are indicated in red, green, and blue letters, respectively. (e) DNA sequence of mutant *Mymk* enhancer. Mutations in the 3 TF binding sites are indicated. (f) Western blot showing the Co-immunoprecipitation of endogenous Myogenin with Klf5 and endogenous Tead4 with Klf5 in C2C12 cells (representative results from 2 independent experiments). Numerical data are presented as mean ± s.e.m. $*P < 0.05$, $**P < 0.01$, $***P < 0.001$, $****P < 0.0001$. For c, unpaired t test for *Myogenin*, *Klf5*, *Tead4*, and One-way ANOVA with adjusted P-value for *Myomaker*, *Col25a1*, *Tnnt2* and *Myomixer*.

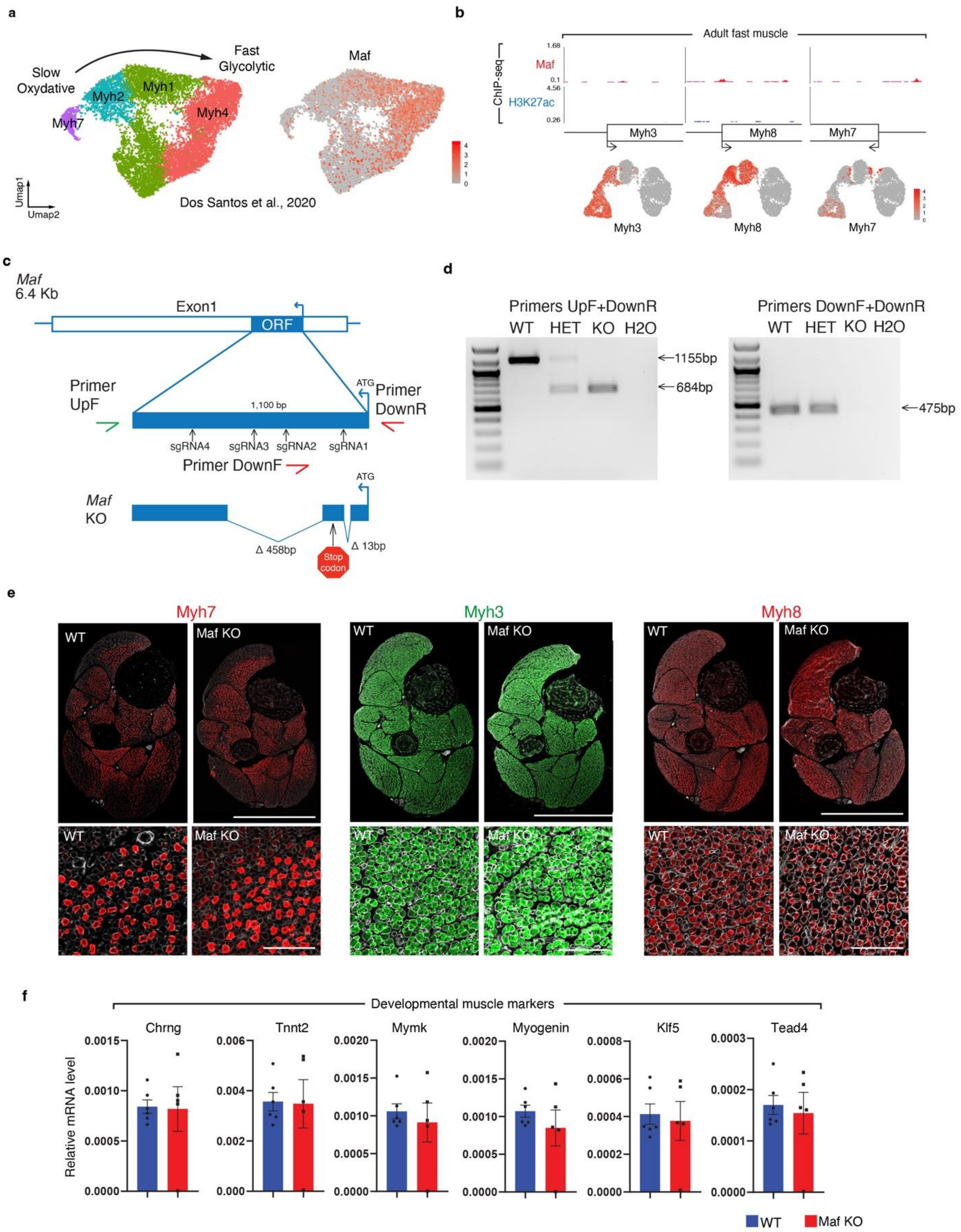

a
Slow
Oxydative
Fast
Glycolytic
Myh2
Myh1
Myh4
Myh7
Maf
Umap1
Umap2
Dos Santos et al., 2020
b
Adult fast muscle
ChIP-seq
Maf
H3K27ac
1.68
0.1
4.56
0.26
Myh3
Myh8
Myh7
c
Maf
6.4 Kb
Exon1
ORF
ATG
Primer
UpF
Primer
DownR
1,100 bp
sgRNA4
sgRNA3
sgRNA2
sgRNA1
Primer DownF
Maf
KO
Δ 458bp
Stop
codon
Δ 13bp
d
Primers UpF+DownR
WT HET KO H2O
1155bp
684bp
Primers DownF+DownR
WT HET KO H2O
475bp
e
Myh7
Myh3
Myh8
WT
Maf KO
f
Developmental muscle markers
Chrng
Tnnt2
Mymk
Myogenin
Klf5
Tead4
Relative mRNA level
WT
Maf KO

Supplementary Fig. 5: Maf activates transcriptionally the maturation of myofibers.

(a) UMAPs depicting Maf mRNA expression in adult fast and slow myonuclei (from Dos Santos et al., 2020). (b) ChIP-Seq tracks depicting the non-binding of Maf in the *Myh3, Myh8, and Myh7* locus. (c) The mouse *Maf* gene showing positions of sgRNAs (sgRNA1, sgRNA2, sgRNA3 and sgRNA4) used for CRISPR-Cas9 mediated knock out of *Maf*. ORF: Open reading frame. (d) Genotyping of mutant animals by PCR, using primers indicated in (c) (representative results from 3 independent experiments). (e) Immunostaining of whole hind limbs of E18.5 WT and Maf KO revealing Myh7 (red) Myh3 (green) and Myh8 (red) positive myofibers (representative results from 3 independent experiments). Scale bar on top images: 1mm. Scale bar on bottom images: 100µm (f) Expression level by RT-qPCR of the developmental genes *Chrng*, *Tnnt2*, *Mymk*, *Myogenin*, *Klf5* and, *Tead4* in WT and Maf KO hindlimb skeletal muscle. The developmental gene program is expressed similarly in control and Maf KO muscles (n=6 different animals for WT, and n=5 different animals for Maf KO). Numerical data are presented as mean ± s.e.m.

a

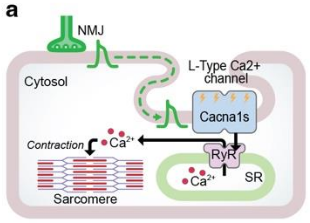

b

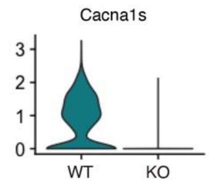

c

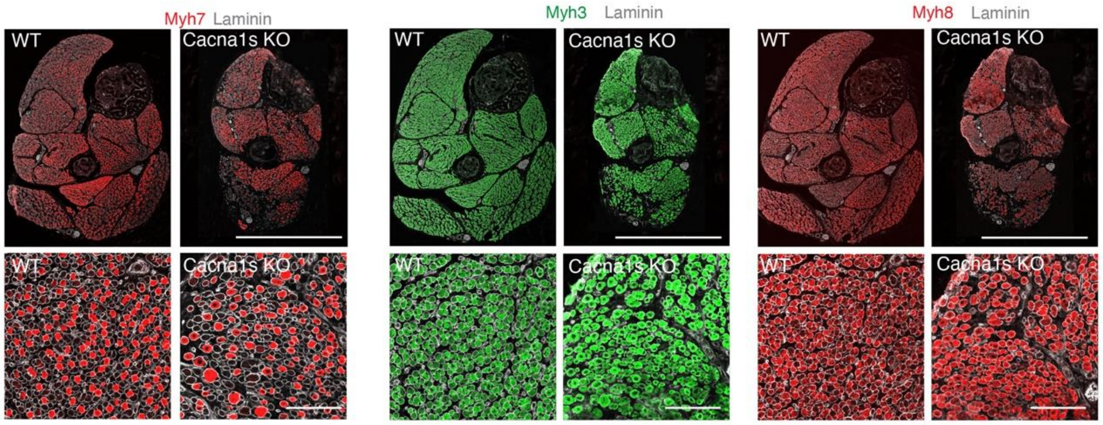

d

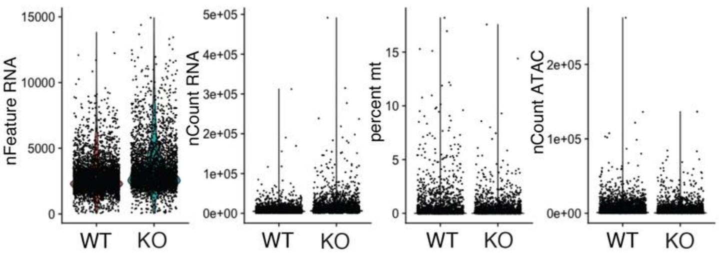

e

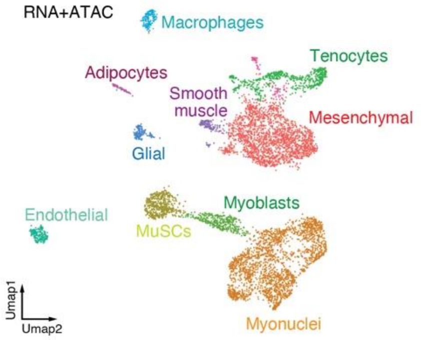

f

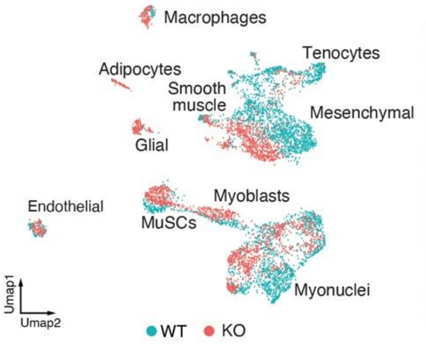

	% WT	% KO
MuSCs	7.5	16.7
Myoblasts	6.1	11.4
Adipocytes	0.4	5.4
Glial	1.6	7.0
Smooth muscle	2.5	4.4
Macrophages	2.4	5.9
Endothelial	4.4	5.1
Mesenchymal	52.0	38.9
Tenocytes	20.6	3.6
Osteoblast	2.5	1.6

g

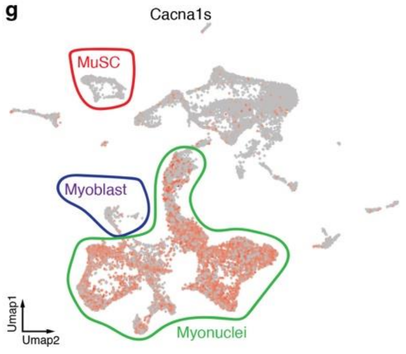

Supplementary Fig. 6: Impairment of myofiber maturation in Cacna1s KO mice and gene expression analysis.

(a) Diagram of the excitation-contraction coupling mechanism in skeletal muscle. CACNA1s is a subunit of the L-type calcium channel complex. (b) Expression level of Cacna1s in WT and Cacna1s KO muscles by snRNA-seq. (c) Immunostaining of whole hind limbs of E18.5 WT and Cacna1s KO revealing Myh7 (red) Myh3 (green) and Myh8 (red) positive myofibers (representative results from 3 independent experiments). Scale bar on top images: 1mm. Scale bar on bottom images: 100µm (d) Number of reads and genes detected in snRNA-seq, mitochondrial genes, reads in snATAC-seq for WT and Cacna1s KO E18.5 hindlimb muscles. (e) UMAP visualization of the nuclei from WT and Cacna1s KO muscles from the integrative analysis of snRNA-seq and snATAC-seq data by WNN. (f) The same UMAP visualization as in d, showing the genotype of each nucleus. On the right: percentage of non-myonuclei in each cluster in WT and Cacna1s KO muscles. (g) The same UMAP visualization as in Fig. 1b, showing the expression of Cacna1s mRNA during myogenesis.

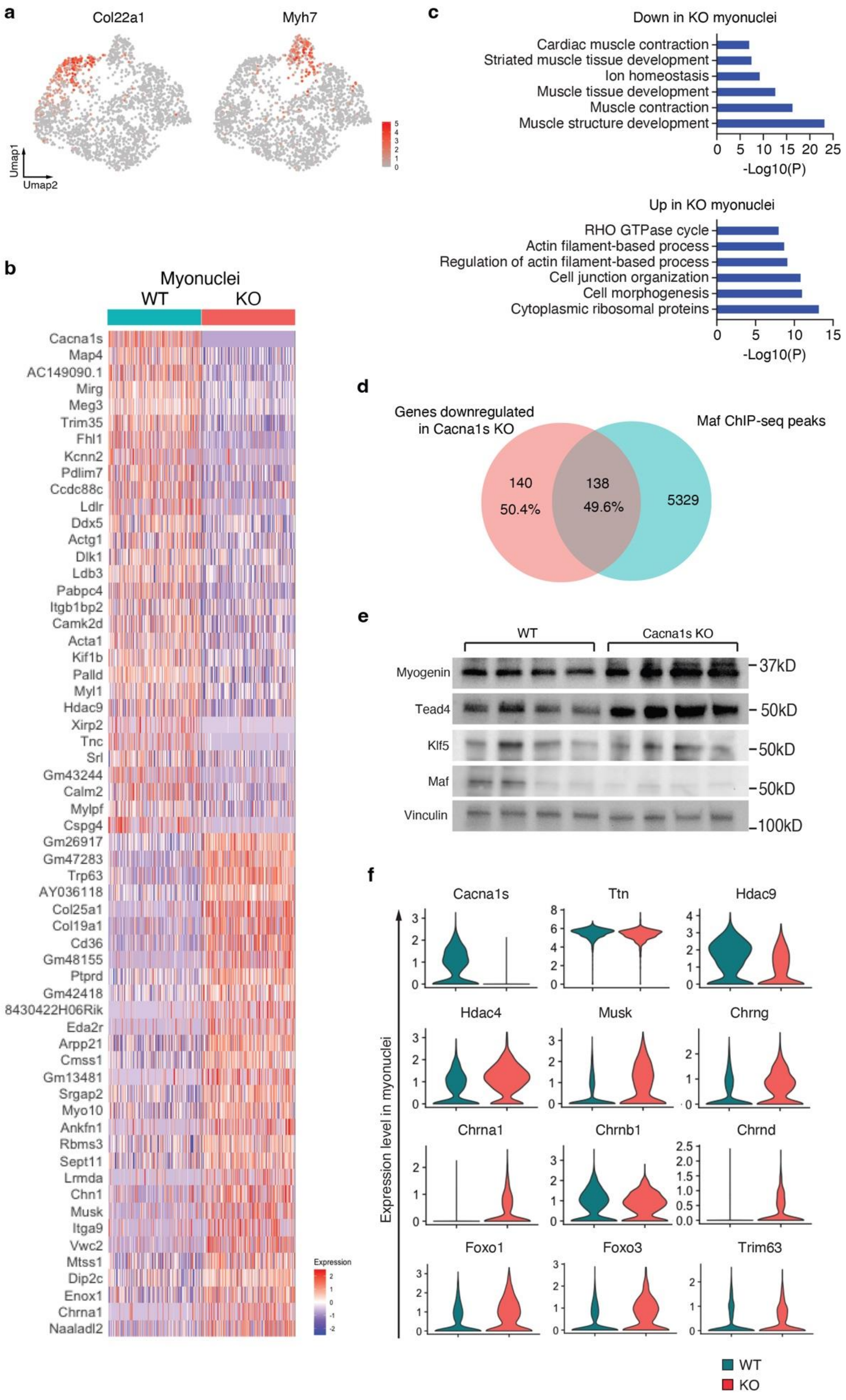
a
Col22a1
Myh7
Umap1
Umap2
b
Myonuclei
WT
KO
Cacna1s
Map4
AC149090.1
Mirg
Meg3
Trim35
Fhl1
Kcnn2
Pdlim7
Ccdc88c
Ldlr
Ddx5
Actg1
Dlk1
Ldb3
Pabpc4
Itgb1bp2
Camk2d
Acta1
Kif1b
Palld
Myl1
Hdac9
Xirp2
Tnc
Srl
Gm43244
Calm2
Mylpf
Cspg4
Gm26917
Gm47283
Trp63
AY036118
Col25a1
Col19a1
Cd36
Gm48155
Ptprd
Gm42418
8430422H06Rik
Eda2r
Arpp21
Cmss1
Gm13481
Srgap2
Myo10
Ankfn1
Rbms3
Sept11
Lrmda
Chn1
Musk
Itga9
Vwc2
Mtss1
Dip2c
Enox1
Chrna1
Naaladl2
Expression
c
Down in KO myonuclei
Cardiac muscle contraction
Striated muscle tissue development
Ion homeostasis
Muscle tissue development
Muscle contraction
Muscle structure development
-Log10(P)
Up in KO myonuclei
RHO GTPase cycle
Actin filament-based process
Regulation of actin filament-based process
Cell junction organization
Cell morphogenesis
Cytoplasmic ribosomal proteins
-Log10(P)
d
Genes downregulated in Cacna1s KO
Maf ChIP-seq peaks
140
50.4%
138
49.6%
5329
e
WT
Cacna1s KO
Myogenin
Tead4
Klf5
Maf
Vinculin
37kD
50kD
50kD
50kD
100kD
f
Cacna1s
Ttn
Hdac9
Hdac4
Musk
Chrng
Chrna1
Chrnb1
Chrnd
Foxo1
Foxo3
Trim63
Expression level in myonuclei
WT
KO

Supplementary Fig. 7: The absence of the L-type Ca2+ channel (Cacna1s KO) led to a suppression of Maf gene expression and myofiber maturation.

(a) The same UMAP visualization as in Fig. 6d, showing the expression of *Col22a1* and *Myh7*. (b) Heatmap of the differentially expressed genes in WT and Cacna1s KO myonuclei (upregulated in red and downregulated in blue). The values correspond to z-scores of normalized counts. (c) Top GO terms enriched in the DEGs in WT and Cacna1s KO myonuclei. (d) Venn diagram representing the overlap of downregulated genes in Cacna1s KO myonuclei that contain Maf ChIP-seq peaks. (e) Western blot showing the expression of Myogenin, Klf5, Tead4, and Maf in WT and Cacna1s KO E18.5 hindlimb skeletal muscle (n=4) (representative results from 2 independent experiments). Vinculin is a loading control. (f) Violin plots showing expression level of several genes in WT and Cacna1s KO myonuclei.

For immunostaining

Target	Species	Antibody reference	Supplier	dilution
Myh7	mouse IgG2B	BA-F8	DHSB	: 1/40
Myh2	mouse IgG1	SC-71	DHSB	: 1/200
Myh4	mouse IgM	BF-F3	DHSB	: 1/200
Myh3	mouse IgG1	BF-45	DHSB	: 1/200
Myh8	mouse IgM	N3-36	DHSB	: 1/200
Laminin	Rabbit	L9393	Sigma	: 1/500

For Western Blot

Target	Species	Antibody reference	Supplier	dilution
Maf	Rabbit	ab77071	Abcam	: 1/1000
Vinculin	Mouse	V9131	Sigma-Aldrich	: 1/1000
Tead4	mouse IgG2a	ab58310	Abcam	: 1/1000
Klf5	Rabbit	21017-1-AP	Proteintech	: 1/1000
Myogenin	mouse IgG1	sc-12732	Santa Cruz Biotechnology	: 1/1000
Parvalbumin	mouse IgG1	MAB1572	Millipore Sigma	: 1/1000
Myoglobin	mouse IgG1	sc-74525	Santa Cruz Biotechnology	: 1/1000
GAPDH	mouse IgG1	MA5-15738	Thermo Fisher Scientific	: 1/1000

Supplementary Table 1: Antibodies used for immunostaining and Western-Blot

Target	Forward	reverse
Maf	CAAGGAGAAATACGAGAAGC	TCACATGAAAAATTCGGGAG
Myh4	GCTTGAAAACGAGGTGGAAA	CCTCCTCAGCCTGTCTCTTG
Actn3	TGCCAAGCCATCTGTGACCAGT	GGTCAATGGTCTCCAGGAGCTT
Mybpc2	GCAGCTTTGTGATCGAGAGTGC	GTCTTCTCCAACTGAGGTGACAC
Ckm	GGCTTCACTCTGGACGTCA	CCTTGAAGACCGTGTAGGACTC
Atp2a1	GAAGCCTCTCTAAAGTGGAGCG	CGTGAGGACTTAGCTGGTGAAC
Chrng	CTTGTGGCTAAGAAGGTGCCTG	GCAAGGACACATTGAGCACGAC
Tnnt2	GCTACAGACTCTGATCGAGGCT	GCTCATTGCGAATACGCTGCTG
Mymk	CAGTGAGCATCGCTACCAAGAG	GAATGTCACGGCGCATGAAGCA
Myogenin	CCATCCAGTACATTGAGCGCCT	CTGTGGGAGTTGCATTCACTGG
Klf5	AGCTCACCTGAGGACTCATACG	AGAAGCTGCGTTGGCACACCAT
Tead4	GCTCTGGATGTTGGAGTTCTCG	TTGGGCTTGACTGGCTGATGTG

Supplementary Table 2: Oligonucleotide primers used for RT-qPCR experiments

Target	Target Sequence	Reference	Supplier
Tead4	GCTGAAACACTTACCCGAGAA	TRCN0000302368	Sigma
Klf5	TCCGATAATTTCAGAGCATAA	TRCN0000055285	Sigma
Myogenin	GAATTTAGCTGACTCCTTAAA	TRCN0000434954	Sigma

Supplementary Table 3: ShRNA used for knock-down experiments

Supplementary figures, uncropped blots

2/17/22

anti-Klf5

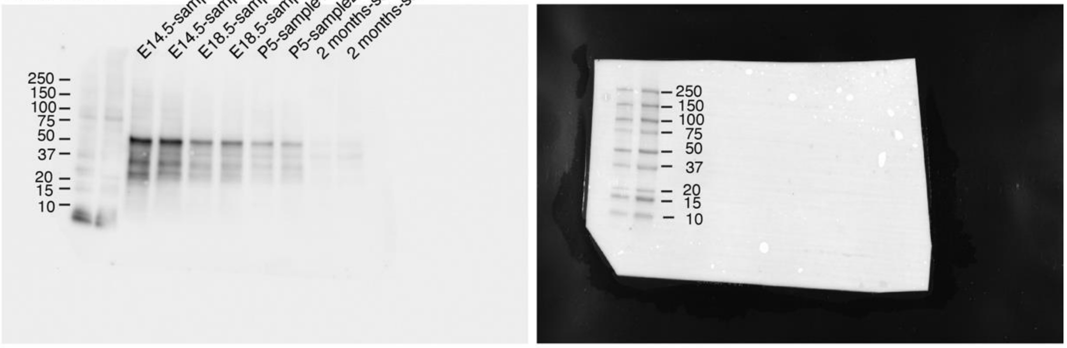

anti-Myogenin

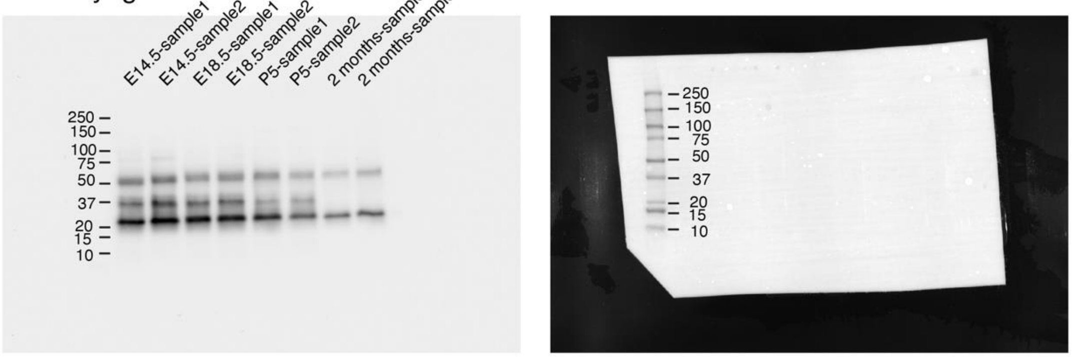

anti-Tead4

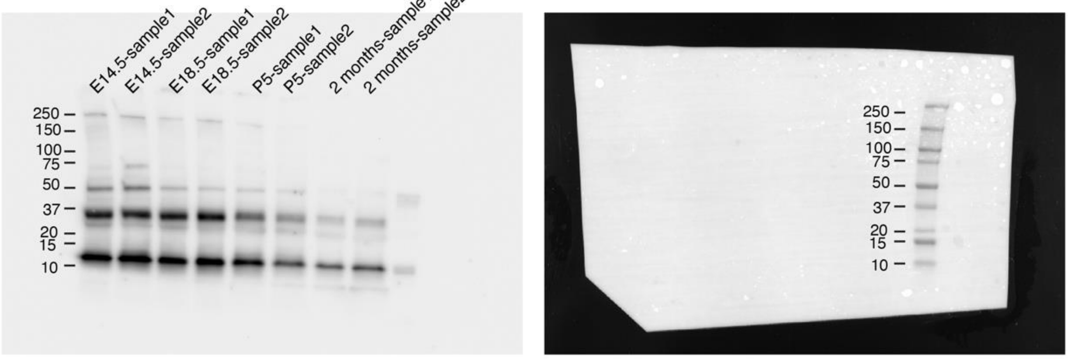

anti-Gapdh

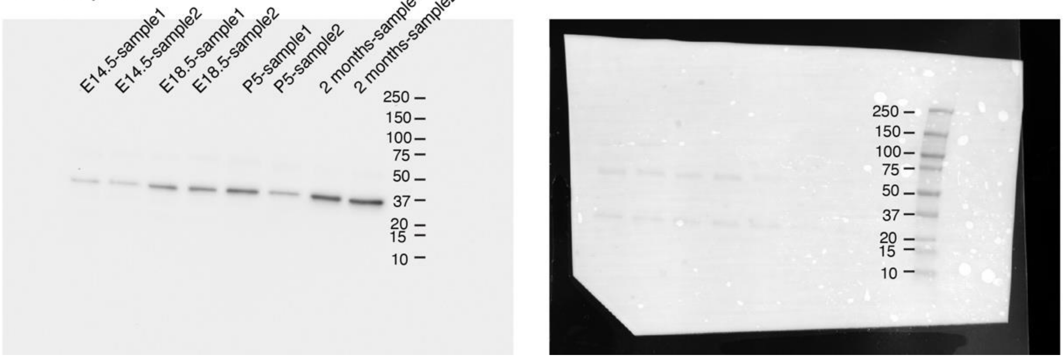

Supplementary figure 4f, uncropped blots

anti-Klf5

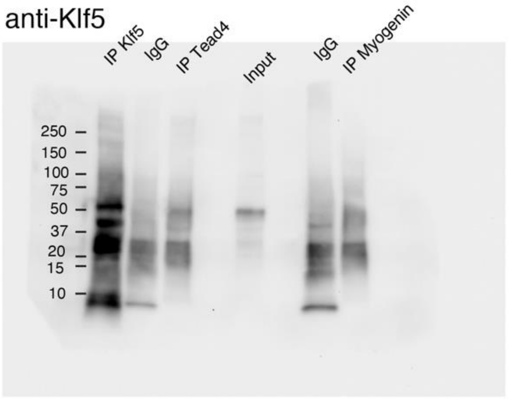

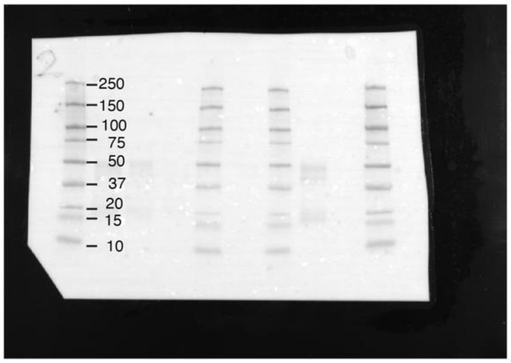

anti-Myogenin

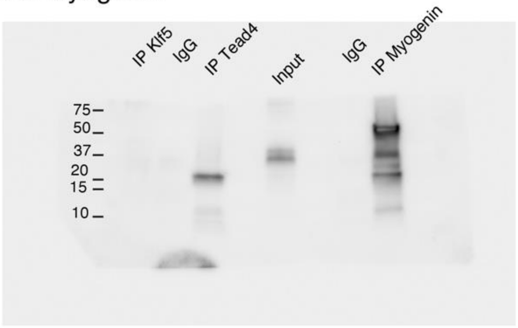

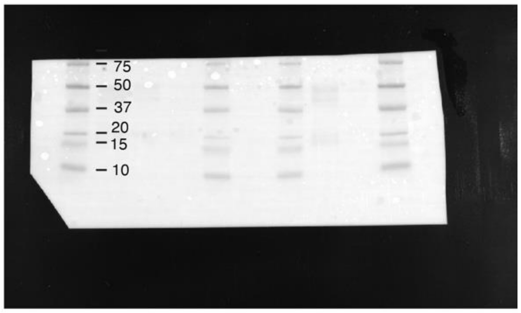

anti-Tead4 low exposure

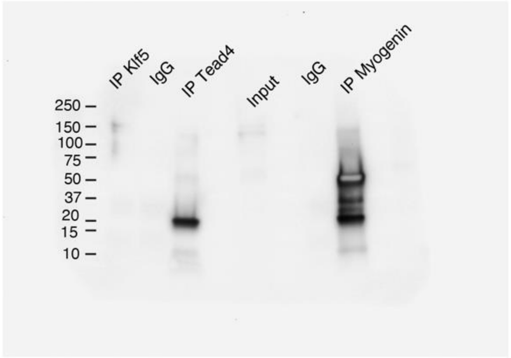

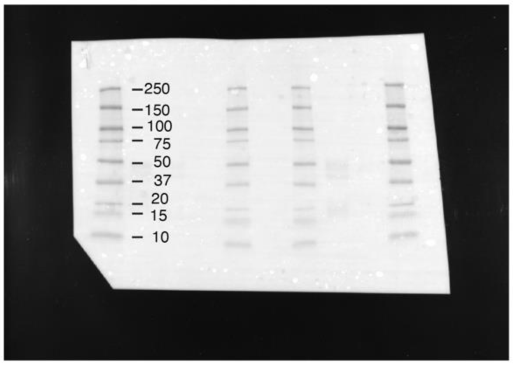

anti-Tead4 middle exposure

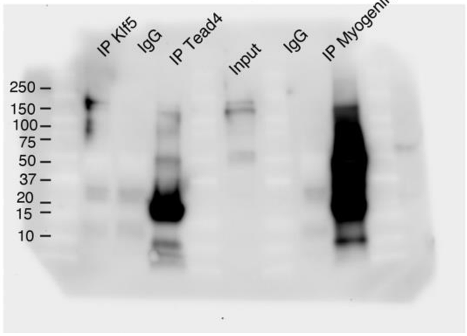

anti-Tead4 high exposure

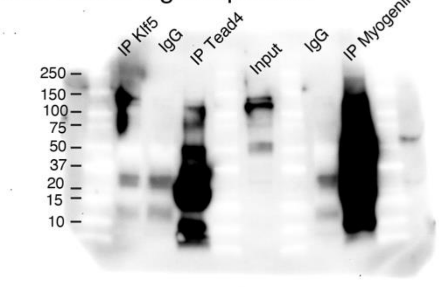

Supplementary figure 4f, uncropped blots

4/05/23

anti-Klf5

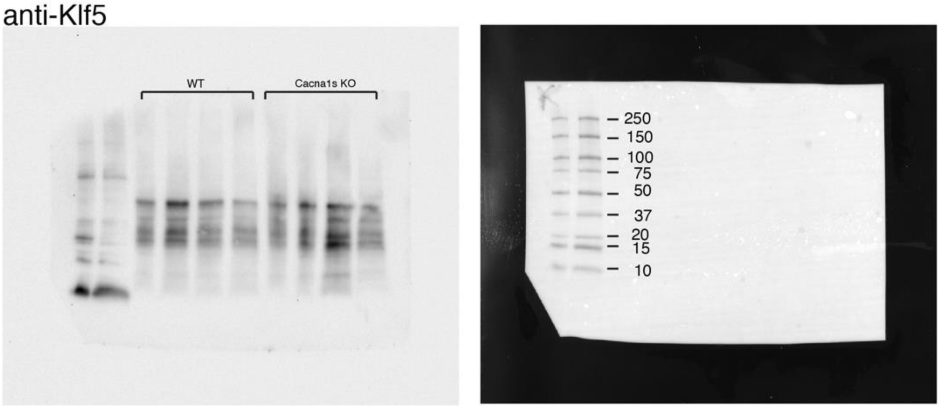

anti-Maf

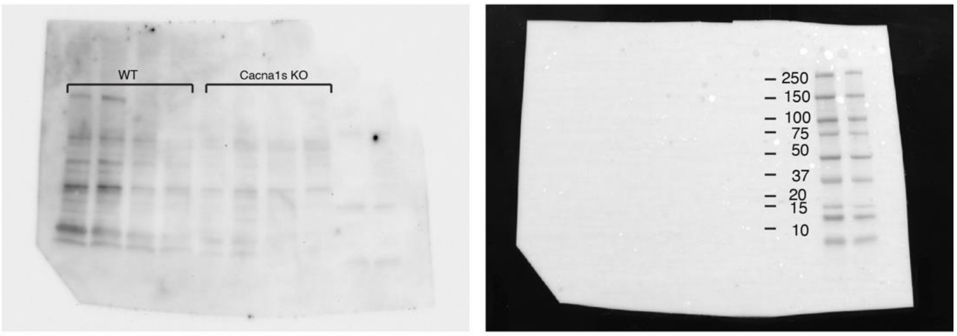

anti-Tead4

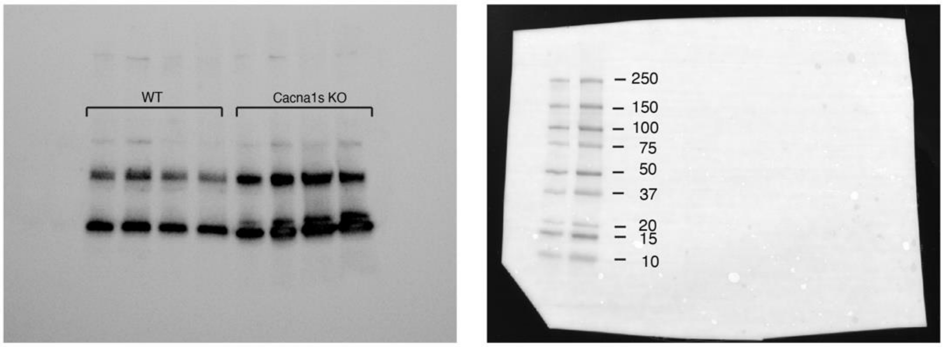

anti-Vinculin

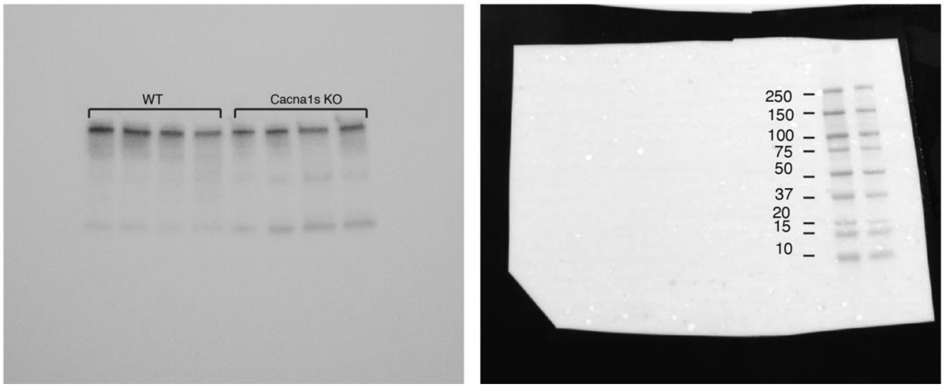

Supplement: Supplementary file 1 — Supplementary Information [file 41467_2023_40073_MOESM1_ESM.pdf]
